# A predictive tool for an effective use of ^18^F-FDG PET in assessing activity of sarcoidosis

**DOI:** 10.1186/1471-2466-12-57

**Published:** 2012-09-14

**Authors:** Rémy LM Mostard, Sander MJ Van Kuijk, Johny A Verschakelen, Marinus JPG van Kroonenburgh, Patty J Nelemans, Petal AHM Wijnen, Marjolein Drent

**Affiliations:** 1 Department of Respiratory Medicine, Atrium Medical Centre, Heerlen, The Netherlands; 2 Department of Epidemiology, University Maastricht, Maastricht, The Netherlands; 3 Department of Radiology, University Hospital Gasthuisberg, Leuven, Belgium; 4 Department of Nuclear Medicine, Maastricht University Medical Centre, Maastricht, The Netherlands; 5 Department of Clinical Chemistry, Maastricht University Medical Centre, Maastricht, The Netherlands; 6 Faculty of Health, Medicine and Life Sciences, University Maastricht, The Netherlands and Department of interstitial lung diseases, Hospital Gelderse Valley, Ede, The Netherlands; 7 Faculty of Health, Medicine and Life Sciences; UNS 40 room 4.550, University Maastricht The Netherlands, PO Box 3100, 6202 NC, Maastricht, The Netherlands

**Keywords:** Clinical prediction rule, High-resolution computed tomography, Soluble interleukin-2 receptor, PET, Sarcoidosis

## Abstract

**Background:**

^18^F-FDG PET/CT (PET) is useful in assessing inflammatory activity in sarcoidosis. However, no appropriate indications are available. The aim of this study was to develop a prediction rule that can be used to identify symptomatic sarcoidosis patients who have a high probability of PET-positivity.

**Methods:**

We retrospectively analyzed a cohort of sarcoidosis patients with non organ specific persistent disabling symptoms (n = 95). Results of soluble interleukin-2 receptor (sIL-2R) assessment and high-resolution computed tomography (HRCT) were included in the predefined model. HRCT scans were classified using a semi-quantitative scoring system and PET findings as positive or negative, respectively. A prediction model was derived based on logistic regression analysis. We quantified the model’s performance using measures of discrimination and calibration. Finally, we constructed a prediction rule that should be easily applicable in clinical practice.

**Results:**

The prediction rule showed good calibration and good overall performance (goodness-of-fit test, p = 0.78, Brier score 20.1%) and discriminated between patients with positive and negative PET findings (area under the receiver-operating characteristic curve, 0.83). If a positive predictive value for the presence of inflammatory activity of ≥90% is considered acceptable for clinical decision-making without referral to PET, PET would be indicated in only 29.5% of the patients. Using a positive predictive value of 98%, about half of the patients (46.3%) would require referral to PET.

**Conclusions:**

The derived and internally validated clinical prediction rule, based on sIL-2R levels and HRCT scoring results, appeared to be useful to identify sarcoidosis patients with a high probability of inflammatory activity. Using this rule may enable a more effective use of PET scan for assessment of inflammatory activity in sarcoidosis.

## Introduction

Sarcoidosis is a multisystemic disease characterized by cellular immunity activity with formation of noncaseating granuloma in various organ systems [1,2]. Assessing the presence of inflammatory activity is important for the management of sarcoidosis and for the follow-up of treatment effect [3-5]. Unlike acute sarcoidosis [6,7], assessment of inflammatory activity in sarcoidosis patients with persistent disabling symptoms that cannot be explained from the results of routine investigations, including the absence of lung functional or chest radiographic deterioration, remains a challenge to clinicians [8-10]. In these patients, it is often complicated to differentiate between reversible and irreversible disease. Symptoms like fatigue can be nonspecific and difficult to objectify [11-14]. Furthermore, symptoms like coughing and dyspnea might be related to ongoing inflammatory activity as well as to end-stage disease, i.e. pulmonary fibrosis. In this respect, it is important to know about the presence or absence of inflammatory activity, as fibrosis itself is irreversible. In general, immunosuppressive treatment does not seems beneficial to patients with fibrosis without ongoing inflammatory activity [11].

Inflammatory activity is characterized by ongoing T-cell and macrophage activity and granuloma formation, reflected by an increase in serological markers of inflammatory activity, i.e. angiotensin-converting enzyme (ACE), soluble interleukin-2 receptor (sIL-2R) and neopterin, or abnormalities of glucose metabolism [7,15,16]. ^18^ F-FDG PET/CT (PET) is used to detect high glucose metabolism and has been shown to be useful for the assessment of inflammatory activity in sarcoidosis [7,17-22]. Previous studies found that elevated serological inflammatory markers, abnormalities on high-resolution computed tomography (HRCT) and impaired lung function were associated with PET-positivity [7,8,23,24]. In addition, diffusely increased metabolic activity in the lung parenchyma was found to predict a future deterioration of diffusion capacity for carbon monoxide (DLCO) when untreated [24].

It is important to gain knowledge and understanding about the appropriate use of this new technique for assessment of inflammatory activity in clinical practice [21,22]. This means that, in view of the radiation dose and costs, defining appropriate indications for PET-scanning is vital. Therefore, the aim of this study was to develop a prediction rule that can be used in clinical practice to identify symptomatic sarcoidosis patients for whom there is a high probability that PET will show the presence of inflammatory activity.

We reviewed the medical records of all sarcoidosis patients referred to the interstitial lung disease service (ild care team) of the department of Respiratory Medicine at the Maastricht University Medical Centre (Maastricht, The Netherlands), a tertiary referral center, between June 2005 and September 2010. The study included all patients who underwent laboratory and lung function testing, HRCT, as well as a PET scan (n = 106). The indication for performing PET was the presence of non organ specific disease-related disabling symptoms persisting for at least one year. Non organ specific persistent disabling symptoms were defined as the presence of more than one symptom that had substantial influence on quality of life, and that could not be explained from the results of routine investigations, including the absence of lung functional or chest radiographic deterioration. The symptoms had to be present for at least one year and included fatigue (Fatigue Assessment Scale [FAS] ≥22) [25], symptoms compatible with small fiber neuropathy (SFN; SFN Screenings List [SFNSL] score ≥11) [26], arthralgia and/or muscle pain, dyspnea (MRC dyspnea scale ≥3), exercise intolerance or coughing. Laboratory and lung function testing were performed within a 2-week interval before or after the HRCT. PET scans were made within a 3-months interval before or after the HRCT, without changing the therapy during this period. The diagnosis was based on consistent clinical features and bronchoalveolar lavage (BAL) fluid analysis results, according to the international guidelines. The diagnosis was confirmed histological, demonstrating noncaseating epitheloid cell granulomas, in most cases (75%) [1]. Patients with known co-morbid conditions associated with positive PET findings were excluded. This meant that five patients with common variable immunodeficiency (CVID), five patients with malignancies and one patient with both rheumatoid arthritis and amyloidosis were excluded. After exclusion based on these criteria, 95 patients were selected. The study protocol was approved by the Medical Research Ethics Committee of the Maastricht University Medical Centre (MEC number 04.145.11).

### Laboratory and lung function tests

Serum levels of sIL-2R were analyzed using commercially available Diaclone ELISA kits (Sanquin, Amsterdam, The Netherlands) and considered elevated if >3154 pg·mL^-1^. Other laboratory analysis and lung function tests were performed as described previously [8].

### Imaging

Thin-section scans with 1-mm collimation were obtained at 10-mm intervals through the chest (Somaton Plus, Siemens, Erlangen, Germany). The scanning parameters included 137 kVP, 255 mA, and 1-s scanning time. Both mediastinal (width 400 HU, level 40 HU) and lung (width 1600 HU, level -800 HU) window images were obtained. Scans were reconstructed with a high-frequency reconstruction algorithm.

A whole body ^18^ F-FDG PET/CT scan was performed using a Gemini® PET/CT (Philips Medical Systems) scanner with time-of-flight (TOF) capability and a 64-slice Brilliance CT scanner. Patients were fasting for at least 6 hours before the examination. In all patients blood glucose was measured to ensure that the blood glucose was below 10 mmol·L^-1^. ^18^ F-FDG (GE Health, Eindhoven, The Netherlands) was injected intravenously and followed by physiologic saline (10 mL). The injected total activity of FDG depended on the weight of the patient. Mean injected dose was: 200 MBq. After a resting period of 45 minutes (time needed for uptake of FDG) PET and CT images were acquired from the head to the feet. A low-dose CT scan was performed without intravenous contrast and was used for attenuation correction of the PET images. The PET images were acquired in 5-minute bed positions. The complete PET data set was reconstructed iteratively with a reconstruction increment of 5 mm to provide isotropic voxel.

### Image analysis

An experienced thoracic radiologist (JV), blinded to the patient’s clinical history and to the PET findings, classified the scans of both lungs using a semi-quantitative HRCT scoring system that has been described by Oberstein et al. [27] and that has been used in previous studies by our group (a detailed description of this scoring system is shown in the Appendix) [28].

All PET scans were interpreted by an experienced nuclear medicine physician (MvK), blinded to the patient’s clinical history and to the HRCT findings. PET findings in the lungs, lymph nodes, or other soft tissues or bones were scored as either positive or negative. A positive PET scan interpretation was performed visually, with a threshold standardized uptake value (SUVmax) ≥2.5. ^18^ F**-**FDG uptake was quantified by drawing a region of interest around the area of pathology of the co-registered transaxial slice. SUVmax was calculated as the maximal pixel activity within the region of interest.

Inter-reader reliability of both the total HRCT score and the simple PET classification system had already demonstrated good agreement in the above-mentioned studies with the same observers (with weighted kappa values of 0.99 and 1.00, respectively) [8,28]. Accordingly, a single radiologist and a single nuclear physician were regarded as sufficient in the present study.

### Potential predictors

Because of the limited number of patients with a negative PET scan (the least frequent outcome in this study) and the usual recommendation to include one predictor for at least ten events, we had to select the two predictors with the strongest associations with PET-positivity [29]. Based on the results of recent studies, the following clinical characteristics were selected in view of their association with PET-positivity: elevated serological inflammatory markers (sIL-2R and neopterin), HRCT abnormalities as assessed by the HRCT scoring system, and lung function tests (forced vital capacity (FVC), DLCO) [8,23]. Of the serological inflammatory parameters, positive sIL-2R had shown the strongest association with PET-positivity in the previous study [8]. Neopterin was not added as predictor since neopterin values were missing in almost half of the studied patients. Moreover, from a practical point of view sIL-2R also is preferable considering that in clinical practice accessibility to neopterin measurement is less compared to sIL-2R. Lung function tests and HRCT scoring results were strongly associated with each other [23] and we decided to include the total HRCT score. In the end, therefore, two potential predictor variables (sIL-2R and total HRCT scoring results) were included in the predefined model.

### Model development

Missing values were imputed using regression imputation, since the omission of patients who have one or more predictor variables missing from the analysis can cause a considerable loss of precision and may bias results [30-32]. Predefined predictors (sIL-2R and the total HRCT score) were entered into a multivariable logistic regression model with the PET result (positive versus negative) as the dependent variable. As recommended by prediction modeling guidelines, the preselected predictors remained in the model irrespective of statistical significance [29].

To assess the performance of the final model, we quantified its performance with respect to discrimination and calibration. Discrimination is the model’s ability to discriminate between PET-negative and PET-positive patients, quantified as the area under the receiver operating characteristic (ROC) curve [33]. This can range from 0.5 (no discrimination) to 1.0 (perfect discrimination). Calibration is used to quantify the agreement between the predicted probability and the actual, or observed, frequencies. To this end, we computed the Hosmer and Lemeshow (H-L) goodness-of-fit statistic. A high H-L statistic will yield a low p-value and provides evidence of lack of fit. As a measure of overall performance, we computed the Brier score, which was scaled from 0 to 100%, so it could be interpreted as an r-squared statistic of explained variance [34]. Statistical analyses were performed using R (version 2.12.2; http://www.r-project.org).

## Results

Table 1 shows a summary of relevant demographic and clinical characteristics of the sarcoidosis patients (n = 95: 87 Caucasians, 5 of African origin and 3 of Asian origin) categorized by the absence (n = 18: 19%) or presence (n = 77: 81%) of positive PET findings. The PET-positive group demonstrated significantly higher sIL-2R levels (n = 77; 18 (18.9%) missing values) and total HRCT scores, but lower DLCO values compared to the PET-negative group. The number of patients with a chest radiography (CXR) stage 0/I was higher in the PET-negative group. In the PET-positive group, 56/77 (73%) patients showed pulmonary PET-positive findings and 61/77 (79%) demonstrated extrathoracic PET-positive findings.

**Table 1:** Clinical and demographic characteristics of the sarcoidosis patients categorized by absence or presence of positive PET findings

	**PET - patients**	**PET + patients**	**p-value**
	**(n = 18)**	**(n = 77)**	
age (yrs)	46 (22-72)	45(24-76)	0.585
sex (male)	11 (61%)	44 (57%)	0.762
time since diagnosis (yrs)	4 (1-20)	2 (1-21)	0.469
Therapy total, number (%)	6 (33%)	20 (26%)	0.461
1/2/3/4	3/0/3/0	10/1/7/2	0.444
ACE (9-25 U/L)	15 (1-29)	18 (3-60)	0.136
sIL-2R (240-3154 pg/mL)	1784 (518-4614)	3434 (1191-15000)	0.002
Neopterin (<2.5 ng/mL)	1.7 (0.8-2.6)	2.8 (0.7-18.2)	0.030
CRP (2-9 μg/mL)	6 (1-15)	6 (1-80)	0.249
CXR stage 0/I	12/1	18/14	0.005
CXR stage II/III/IV	1/3/1	12/8/25	0.449
Total HRCT score	2.9 ± 3.0	6.0 ± 3.9	0.002
FVC total (% pred)	90 ± 22	91 ± 22	0.818
CXR I-II	94 ± 24	103 ± 15	0.174
CXR II-IV	79 ± 14	83 ± 22	0.741
DLCO total (% pred)	78 ± 18	69 ± 20	0.046
CXR I-II	79 ± 17	80 ± 17	0.888
CXR II-IV	71 ± 19	60 ± 19	0.234

Data are presented as median with range in parentheses; mean ± SD; absolute numbers or percentages if appropriate. PET: positron emission tomography; -: negative; +: positive; n:number; yrs: years; therapy total: total number of patients treated at time of PET scanning; 1: prednisone monotherapy; 2: methotrexate monotherapy; 3: prednisone and methotrexate combination therapy; 4: methotrexate and infliximab combination therapy; ACE: serum angiotensin-converting enzyme; sIL-2R: soluble interleukin-2 Receptor; CRP: C-reactive protein; CXR: chest radiography; HRCT: high-resolution computed tomography; FVC: forced vital capacity; % pred: percentage of predicted values; DLCO: diffusion capacity for carbon monoxide. p < 0.05 was considered to indicate significance.

Signs of fibrosis on HRCT were present in 26 patients. Twenty-two (85%) of these patients had positive pulmonary PET findings whereas only four (15%) had negative pulmonary PET findings (p = 0.002). Median SUVmax in the former patients was 7.1 (3.1-16.2). Extrathoracic PET-positive findings were present in 18 (82%) and positive serological inflammatory marker testing in 16 (73%), respectively, of those pulmonary PET-positive patients with signs of fibrosis on HRCT.

### Derivation of the scores in the prediction rule

**Figure 1:**
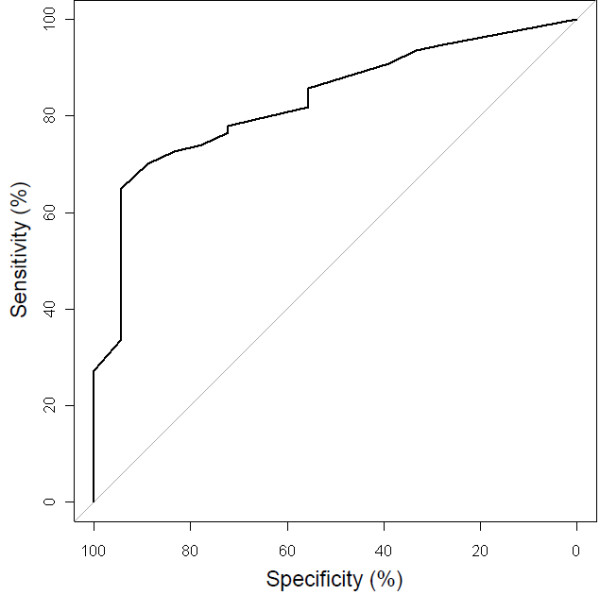
Receiver operating characteristic (ROC) curve of the model’s ability to discriminate between PET-negative and PET-positive patients.

Table 2 shows the regression coefficients and odds ratios as derived from the original prediction model for the probability of a positive PET result, and a validated model after the bootstrap validation. The bootstrap validation yielded a shrinkage factor of 0.93, which was used to adjust the regression coefficients for overfitting. The formula in Table 2 allows predicted probabilities to be calculated for future patients. Figure 1 shows the ROC curve of the internally validated model. The area under the curve was 0.83 (95% confidence interval (CI) = 0.74 – 0.93) indicating good discriminatory ability. Calibration as quantified using the H–L goodness-of-fit test yielded a p-value of 0.78, and the Brier score was 20.1%, indicating both good calibration and good overall performance.

**Table 2:** Regression coefficients and odds ratios with 95% confidence intervals as derived from the original model and the internally validated model

	**Original model**			**Model after internal validation**	
**Variable**	**Regression coefficient**	**Odds ratio (95% CI)**	**p-value**	Regression coefficient*	**Odds ratio (95% CI)**
Intercept	-0.32	-	-	-0.23	-
Elevated sIL-2R levels	1.98	7.27 (1.86 – 28.40)	0.004	1.85	6.33 (1.73-28.17)
Total HRCT score	0.24	1.27 (1.06 - 1.52)	0.011	0.22	1.25 (1.04-1.50)
To calculate the absolute risk of a positive PET result: P_(PET-positive)_ = (1 / (1 + exp(-(-0.23 + 1.85 * sIL-2R + 0.22 * HRCT score)))) * 100%					

CI: confidence interval; sIL-2R: soluble interleukin-2 Receptor; HRCT: high-resolution computed tomography; PET: positron emission tomography.

^*^ Regression coefficients after adjustment for overfitting by shrinkage (shrinkage factor = 0.93); the intercept was re-estimated.

To improve feasibility of the prediction rule in clinical practice, the rescaled regression coefficients for sIL-2R and the total HRCT score were multiplied by 4 in order to arrive at scores of 8 for a positive sIL-2R result and 1 per unit of the HRCT score, respectively. Hence, the prediction rule score is based on sIL-2R results (0 points for a negative result and 8 points for a positive result) and total HRCT score (0-18 points). For example, the prediction rule score for a patient with a positive sIL-2R result and a total HRCT score of 5 would be 13 (8 + 5) points.

The implications of using the prediction rule in clinical practice are shown in Table 3. The right column displays the number and percentage of patients that would have an indication to be referred for PET using consecutive cut-off points for the prediction rule score. No data above a prediction rule score of 21 points are displayed, since this was the highest prediction score observed in the included patients. As this table shows, a prediction rule score of ≥6 points (which can be attained with a positive sIL-2R result only) is associated with a positive predictive value of ≥91.0% for the presence of inflammatory activity, whereas the negative predictive value would be 42.9% in this case. If a positive predictive value of ≥90% is considered acceptable for clinical decision-making without referral to PET, only patients with a score <6 (29.5% (28/95)) would have to be referred for PET (Table 3, right column). Using a somewhat higher cut-off value of ≥10 points yields a positive predictive value ≥98%, and about half of the patients (46.3%) would require referral to PET.

**Table 3:** Sensitivity, specificity, and positive and negative predictive values for PET-positivity at consecutive cut-off points of the prediction rule score

Prediction rule cut-off point*	Sensitivity (%)**	Specificity (%)**	Positive predictive value (%)**	Negative predictive value (%)**	Patients referred for PET (%)***
0	100 (77/77)	0 (0/18)	81 (77/95)	-	0 (/95)
1	94.8 (73/77)	27.8 (5/18)	84.8 (73/86)	55.5 (5/9)	9.5 (9/95)
2	93.5 (72/77)	33.3 (6/18)	85.7 (72/84)	54.5 (6/11)	11.6 (11/95)
3	90.9 (70/77)	38.9 (7/18)	86.4 (70/81)	50.0 (7/14)	14.7 (14/95)
4	85.7 (66/77)	55.6 (10/18)	89.2 (66/74)	47.6 (10/21)	22.1 (21/95)
5	81.8 (63/77)	55.6 (10/18)	88.7 (63/71)	41.7 (10/24)	25.3 (24/95)
6	79.2 (61/77)	66.7 (12/18)	91.0 (61/76)	42.9 (12/28)	29.5 (28/95)
7	77.9 (60/77)	72.2 (13/18)	92.3 (60/65)	43.3 (13/30)	31.6 (30/95)
8	76.6 (59/77)	72.2 (13/18)	92.2 (59/64)	41.9 (13/31)	32.6 (31/95)
9	72.7 (56/77)	83.3 (15/18)	94.9 (56/59)	41.7 (15/36)	37.9 (36/95)
10	64.9 (50/77)	94.4 (17/18)	98.0 (50/51)	38.6 (17/44)	46.3 (44/95)
11	53.2 (41/77)	94.4 (17/18)	97.6 (41/42)	32.0 (17/53)	55.8 (53/95)
12	48.1 (37/77)	94.4 (17/18)	97.4 (37/38)	29.8 (17/57)	60.0 (57/95)
13	41.6 (32/77)	94.4 (17/18)	97.0 (32/33)	27.4 (17/62)	65.3 (62/95)
14	35.1 (27/77)	94.4 (17/18)	96.4 (27/28)	25.4 (17/67)	70.5 (67/95)
15	27.3 (21/77)	100 (18/18)	100 (21/21)	24.3 (18/74)	77.9 (74/95)
16	24.6 (19/77)	100 (18/18)	100 (19/19)	23.7 (18/76)	80.0 (76/95)
17	20.8 (16/77)	100 (18/18)	100 (16/16)	22.8 (18/79)	83.2 (79/95)
18	13.0 (10/77)	100 (18/18)	100 (10/10)	21.2 (18/85)	89.5 (85/95)
19	7.8 (6/77)	100 (18/18)	100 (6/6)	20.2 (18/89)	93.7 (89/95)
20	7.8 (6/77)	100 (18/18)	100 (6/6)	20.2 (18/89)	93.7 (89/95)
21	3.9 (3/77)	100 (18/18)	100 (3/3)	19.6 (18/92)	96.8 (92/95)

*Patients were considered test-positive if the prediction rule score was at or above this level.

**Data in parentheses represent proportions.

***Number of patients that would have been indicated for referral to PET if the prediction rule score was at or above the corresponding level.

**Figure 2:**
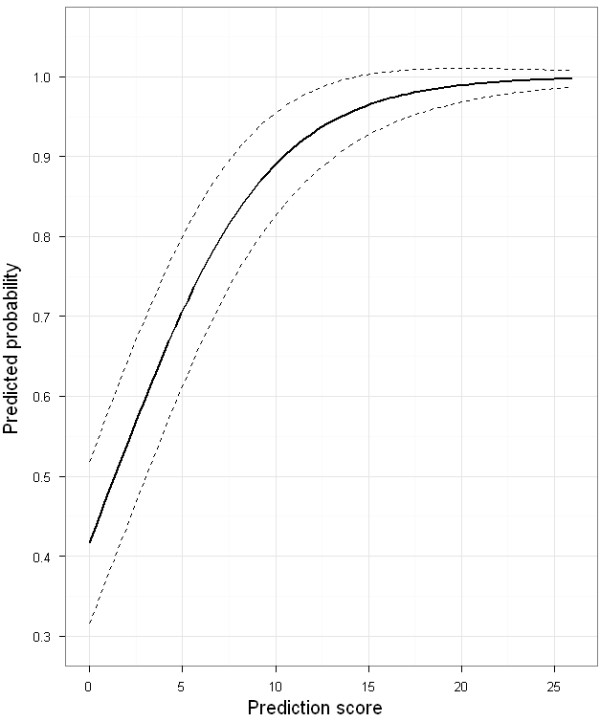
Predicted probability of inflammatory activity being detected by PET in sarcoidosis patients as a function of the prediction rule score.

Figure 2 provides a graphical representation of the predicted values and their 95% CIs. The probability can also be calculated using the formula given in Table 2.

## **Discussio**n

In the present study, we developed an internally validated clinical prediction rule that appeared to be useful to identify symptomatic sarcoidosis patients in whom the presence of inflammatory activity is highly likely. This clinical prediction rule is based on sIL-2R (positive/negative) and HRCT results. Inflammatory activity was regarded as present if PET findings were positive.

To the best of our knowledge, this is the first clinical prediction rule to predict inflammatory activity in sarcoidosis patients that has been constructed according to the methodological standards for clinical prediction rules.

In general, assessment of inflammatory activity is vital in the management of sarcoidosis, and is especially necessary to monitor the course of sarcoidosis and guide therapeutic strategies [3-5]. The presence of inflammatory activity is considered to indicate persistent evolution of the disease and may therefore be a target for therapy. The presence of inflammatory activity can be regarded as certain in case CXR findings or lung function test results provide evidence of disease progression [4]. However, the management of patients with non organ specific persistent disabling symptoms requires reliable and clinically useful markers of inflammatory activity. PET has been shown to be a very sensitive technique to assess inflammatory activity in sarcoidosis [7,17-20]. Several reports demonstrated a significant reduction of FDG uptake after the initiation or modification of treatment in sarcoidosis patients [5,17,18,35,36]. Keijsers et al. [5] demonstrated that changes in PET imaging in a small cohort of sarcoidosis patients treated with infliximab correlated with clinical improvement. Another study showed that diffuse pulmonary parenchymal activity in sarcoidosis patients, as imaged by ^18^ F-FDG PET, predicted a future deterioration of DLCO when medical treatment was withheld, while treatment significantly improved lung function [24].

In the present study, a high frequency of both pulmonary- and extrathoracic PET-positivity as well as serological signs of inflammatory activity was established in the majority of patients. Even most sarcoidosis patients with signs of pulmonary fibrosis demonstrated positive pulmonary PET findings and extrathoracic PET-positive findings (82%) and increased serological inflammatory markers (73%). These findings strongly suggest that PET-positive findings in sarcoidosis patients with CXR stage IV are related to inflammatory activity.

This supports the value of PET, even in patients with signs of pulmonary fibrosis [8,23]. Deciding which sarcoidosis patients with signs of pulmonary fibrosis may benefit from pharmacological treatment remains a challenge to clinicians, as it is not always clear whether respiratory symptoms in these patients are a result of organ damage or due to ongoing inflammation or both. To date, there is no medication with the capability of reversing fibrosis, but treatment might arrest fibrosis of reversible granulomas that persist among the fibrotic elements [37]. Teirstein et al. [17] reported a response to therapy in PET-positive patients, including patients with radiographic stage IV, with partial clearing of the parenchymal radiographic abnormalities, improvement in pulmonary function and decreased hypermetabolism by repeated PET. Furthermore, detecting extrathoracic inflammatory lesions may also provide an explanation for (mainly extrathoracic) symptoms [8].

The question can be raised which patients might benefit from having a PET scan [21,22]. Until now, there have been no guidelines for selecting those patients with non organ specific persistent disabling symptoms for whom PET might offer added value in the assessment of inflammatory activity. The prediction rule developed in the present study proved to be able to distinguish between patients with positive and negative PET findings. Applying this simple prediction rule quantifies the probability that inflammatory activity will be established by PET. As presented in Table 3, PET would only be indicated in less than one third of the patients in our study if a positive predictive value for the presence of inflammatory activity (PET-positivity) of ≥90% is considered acceptable for clinical decision making without referral to PET. This predictive value was reached with a positive sIL-2R result alone (8 points; positive predictive value 92.2%). Among patients with a normal sIL-2R level, PET appeared only to be indicated in those with a total HRCT score of <6 points (positive predictive value <90%). If a positive predictive value of approximately 100% is considered desirable, a cut-off value of <10 points can be used (positive predictive value <98%) in deciding whether to refer patients to PET. In this case, referral to PET would be indicated in about half of the patients.

Using this prediction rule to select patients for whom a PET might be indicated can help to reduce the number of PET scans. This may result in considerable cost reductions, as only a limited number of tests have to be used and these tests are far less expensive than PET. In addition, our prediction rule represents the first attempt to standardize the assessment of inflammatory activity in sarcoidosis.

It should be noted that this rule has been developed in a sample of sarcoidosis patients with non organ specific persistent disabling symptoms. It was derived from and validated in patients from a single referral centre for sarcoidosis, which could limit the generalization of these results. Hence, validation of this prediction rule in other, and larger, sarcoidosis patient populations is warranted. We used bootstrap validation for the internal validation, and adjusted the regression coefficients using the shrinkage factor. Prospective evaluation of the prediction rule in the future should use the internally validated model as described in Table 2.

In conclusion, the derived and internally validated clinical prediction rule, based on sIL-2R levels and HRCT scoring results, appeared to be useful to identify sarcoidosis patients with a high probability of inflammatory activity. Hence, using this rule may be helpful to identify sarcoidosis patient in whom a PET might be of additional value to assess inflammatory activity. These results may affect patient care by providing supportive evidence for more effective use of PET scan in the assessment of inflammatory activity in sarcoidosis.

## Abbreviations

ACE: Angiotensin-converting enzyme; BAL: Bronchoalveolar lavage; CI: Confidence interval; CVID: Common variable immunodeficiency; CXR: Chest radiography; DLCO: Diffusion capacity for carbon monoxide; FAS: Fatigue assessment scale; ^18^F-FDG: Fluorine18-fluorodeoxyglucose; FVC: Forced vital capacity; H-L: Hosmer and Lemeshow; HRCT: High-resolution computed tomography; PET/CT: Positron emission tomography/computed tomography; sIL-2R: Soluble interleukin-2 receptor; ROC: Receiver operating characteristic; SFN: Small fiber neuropathy; SFNSL: Small fiber neuropathy screenings list; SUV: Standard uptake value ; TOF: Time-of-flight.

## Competing interests

The authors declare that they have no competing interests.

## Authors’contributions

RM has made significant contributions to the conception and design of the study, selected data of eligible study subject, has contributed to the interpretation of the data, prepared the draft version of the article and has critically revised the article for important intellectual content. SvK and PN have made substantial contributions to conception and design, analysis and interpretation of data, and revised the article for important intellectual content. JV and MvK have made significant contributions to the acquisition of data, analysis and interpretation of data, and revised the article for important intellectual content. PW has selected data of eligible study subject and has critically revised the article for important intellectual content. MD has made significant contributions to the conception and design of the study, selected data of eligible study subject, has contributed to the interpretation of the data, and has critically revised the article for important intellectual content. All authors read and approved the final approval of the manuscript.

## Pre-publication history

The pre-publication history for this paper can be accessed here:

http://www.biomedcentral.com/1471-2466/12/57/prepub
